# Spontaneous intratumoral bleeding and rupture of giant gastric stromal tumor (> 30 cm) in a young patient

**DOI:** 10.1186/1477-7819-6-76

**Published:** 2008-07-15

**Authors:** Ruy J Cruz, Rodrigo Vincenzi, Bernardo M Ketzer, Andre L Cecilio, Lourdes A Cepeda

**Affiliations:** 1 Department of Surgery, University of Santo Amaro Medical School, Sao Paulo, Brazil; 2 Department of Pathology, University of Santo Amaro Medical School, Sao Paulo, Brazil

## Abstract

**Background:**

Few cases of GIST bigger than 15 cm have been reported in medical literature, all primarily in elderly patients. We report an unusual case, in which a giant gastric GIST – in a young patient – presented as spontaneous intratumoral bleeding followed by intraluminal rupture.

**Case presentation:**

A 37-year-old man was admitted with an acute onset of abdominal pain. CT showed a 32 × 25 cm mass with some cystic lesions and areas of calcification. Twelve hours after admission the patient presented with an episode of upper GI bleeding, and a significant decrease of tumor size and hemoglobin level. An upper endoscopy showed a large bulge in the posterior aspect of the gastric wall, and a small ulcer with continuous bleeding coming from a central orifice. A subtotal gastrectomy was carried out. Pathological examination showed a giant gastric GIST measuring 32 × 25 × 21 cm and weighing 3.750 g. Immunohistochemical staining demonstrated positive reactivity to C-kit protein, CD34, and α-smooth muscle actin; but negative reactivity to S-100 protein.

**Conclusion:**

Intratumoral bleeding is a very rare presentation of GIST; preoperative diagnosis is always made difficult by the absence of pathognomonic signs or symptoms. Emergency local excision with negative margins associated with adjuvant therapy with imatinib mesylate remains the main modality of treatment for high risk GISTs.

## Background

Gastrointestinal stromal tumor (GIST) is a generic name for a mesenchymal tumor originating in the muscular wall of hollow viscera that expresses the c-kit proto-oncogen protein [1-3]. Most GISTs are larger than 5 cm in diameter at time of diagnosis, with a diameter of 10 cm being associated with a higher risk of local or distant metastasis. Few cases of GIST bigger than 15 cm have been reported in medical literature, all of them in elderly patients. Gastrointestinal bleeding is the most common presentation (50%) of GISTs and is usually associated with a ulceration of the tumor into the lumen [4,5].

We report an unusual case, in which a giant gastric stromal tumor (diameter >30 cm) is presented in a young patient as spontaneous intratumoral bleeding followed by intraluminal rupture.

## Case presentation

A 37-year-old man was admitted to the emergency department of the University of Santo Amaro Medical Center with symptoms of abdominal pain. The patient reported a progressive fatigue and increase of abdominal girth for the last 5 months. The day before his admission the patient reported nausea, vomiting, and vague but persistent abdominal pain. Upon physical examination the patient was fully conscious and alert with a heart rate of 100 beats/min, a blood pressure of 115/65 mmHg, afebrile, and slightly malnourished. Abdominal examination revealed abdominal distension with a firm 30 × 30 cm palpable mass in the upper abdomen with minimal intrinsic mobility. Bowel sounds were hypoactive and rectal examination revealed an empty ampulla. Blood tests included a hemoglobin level of 8.4 g/dL, a white blood cell count of 12.800/mm^3 ^(4 band and 70 segmented neutrophils), platelets of 495.000/μL. Coagulogram, liver function tests, creatinine, and BUN were normal.

**Figure 1:**
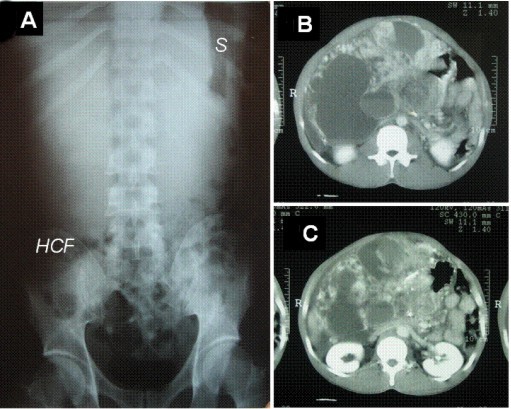
**Radiology**: A) Abdominal X-ray showing a diffuse opaque area in upper abdomen without a gastrointestinal gas shadow, hepatic colon flexure was dislocated downward (HCF), and the stomach (S) to the left side.**B **and** C)**. Abdominal computed tomography scan showing heterogenous mass occupying most part of the abdominal cavity.

An abdominal X-ray showed a diffuse opaque area in the upper abdomen without a gastrointestinal gas shadow; hepatic colon flexure was dislocated downward, and the stomach to the corner of the abdomen (Figure 1A). A CT-scan was performed on the patient which showed a 32 × 25 cm mass with a central thickened septa like enhancing solid component, some cystic lesions, and areas of calcification in the tumor (Figure 1B and 1C).

Twelve hours after admission the patient presented with an episode of upper gastrointestinal (GI) bleeding, a significant reduction of the palpable mass to 25 × 25 cm, and a decrease of the patient's hemoglobin level to 6.4 mg/dl. Fluid resuscitation was started immediately and the patient was transfused with two units of red blood cells. After hemodynamic stabilization an upper endoscopy was performed, showing a large bulge in the posterior aspect of the gastric wall with a small ulcer (1 cm in diameter) with edematous margins in the posterior part of the antrum, and significant bleeding coming from a central orifice.

**Figure 2:**
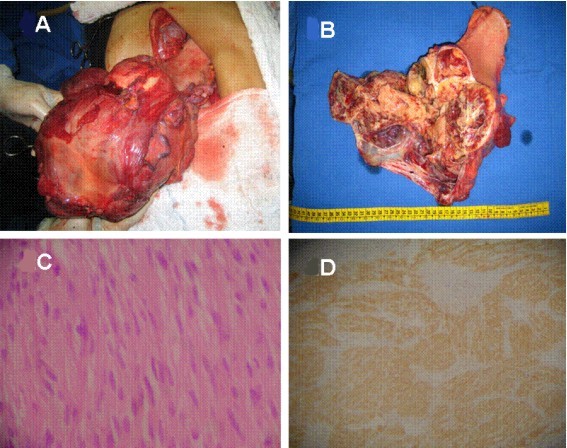
**Clinical photographs and photomicrographs**. **A) **A large, cerebroid and hypervascularized mass occupied the upper abdomen. The tumor arises from the distal part of stomach. **B)**. The ressected mass: a well-circumscribed tumor measuring 30 × 25 × 21 cm and weighting 3.750 g. Cut sections demonstrated a pink, gray and fish-flesh appearance solid parenchyma with focal areas of necrosis, and several blood-filled cysts. **C)**. Microscopically, the tumor was characterized by spindle-shaped tumor cells with acidophilic cytoplasm surrounded by a well-defined cell membrane (Hematoxylin & Eosin ×200). **D)**. Immunohistochemical staining of the tumor tissue demonstrates positive reactivity to c-kit.

Under the diagnosis of persistent GI bleeding an emergency laparotomy was performed. A large cerebroid and hypervascularized mass occupying the entire abdominal cavity was observed, arising from the distal part of the stomach. No other significant findings such as ascites or locoregional metastasis were found at this time. A subtotal gastrectomy and omentectomy was carried out, and the tumor was completely ressected (Figure 2A). The surgical margins were then found to be tumor free.

The ressected mass was a well-circumscribed tumor weighting 3.750 g and measuring 32 × 25 × 21 cm. Cut sections demonstrated a pink, gray, and fish-flesh appearance. The solid parenchyma had focal areas of necrosis, and several blood-filled cysts (Figure 2B). The small ulcer located in the posterior aspect of the gastric wall presented a communication with the larger cystic cavity.

Microscopically, spindle-shaped tumor cells with acidophilic cytoplasm surrounded by a well-defined cell membrane were growing externally from the gastric muscular propria. Nuclear atypia was prominent with mitotic activity of 10 mitoses/50 high-power field. Immunohistochemical staining demonstrated positive reactivity to C-kit protein, CD34, and α-smooth muscle actin, but negative reactivity to S-100 protein.

The patient had an uneventful postoperative course and was discharged on the eighth postoperative day. The patient was put on imatinib mesylate 400 mg daily, and followed up in the outpatient clinic without any signs of recurrence 12 months after surgery.

## Discussion

Gastrointestinal stromal tumor (GIST) is a generic name for a mesenchymal tumor originating in the muscular wall of a hollow viscera that expresses the c-kit proto-oncogen protein. The expression of this protein distinguishes GIST from the true leiomyomas, leiomyoblastomas, and other mesenchymal tumors of the GI tract [1-3,6,7]. Most gastrointestinal mesenchymal tumors belong to the category of GIST; except in the esophagus where typical leiomyomas are more common than GISTs [5,6].

In general, tumor size (> 5 cm) and mitotic index (> 5/50 high-power field) are accepted as two independent prognostic factors of gastrointestinal stromal tumor [5]. Carrillo et al reported that MIB-1 index (> 22% in the most active area) was the most powerful predictor of poor survival [8]. Following these two classification our case is classified as high risk with a poor prognosis. However, no signs of recurrence were observed 12 months after the tumor resection. Although, tumor recurrence has been reported as long as 30 years after primary resection [9,10].

Transarterial embolization could be a reasonable alternative to control the GI bleeding in patients with GIST. However, we decided to perform exploratory laparotomy for two reasons. First, the patient presented a significant drop of hemoglobin levels (8.5 to 6.9 mg/dl) requiring aggressive fluid resuscitation and blood transfusion in order to maintain hemodynamic stability. Secondly, the size of the tumor and the fact that the upper endoscopy showed a continuous and profuse intraluminal bleeding, which would prevent the utilization of transarterial embolization.

Several ongoing clinical trials have been designed to investigate the use of imatinib mesylate as adjuvant therapy for resectable GIST (MDACC, ID03-0023, RTOG-S0132). Recently deMatteo *et al*., evaluated 708 patients who underwent complete gross resection of a primary GIST. This study did demonstrate that imatinib increases recurrence free survival (97% *vs. *83%; imatinib 400 mg daily for 1 year *vs. *placebo). However, no differences on patient survival were observed in this study (ACOSOG Z9001). Therefore, some authors suggest that adjuvant therapy should be consider for at least 1 year in patients with intermediate or high risk [11,12].

GIST usually presents as gastrointestinal bleeding or a palpable abdominal mass. Other rare presentations include bowel or biliary obstruction, dysphagia, abdominal pain, intussusception, and hypoglycemia [3,5,13-15]. Bleeding is usually caused by tumor ulceration at the mucosal level, and usually requires an immediate surgical approach to control the GI bleeding.

Spontaneous rupture of gastric GIST occurs infrequently, and the most common site of rupture of these tumors is the GI lumen [13]. However, rupture of the peritoneal cavity causing massive intraabdominal bleeding and peritonitis were also reported [16-18]. Recently, John et al have reported an unusual case of gastric GIST presented as intratumoral bleeding documented by angiography [19].

We herein report a rare presentation of intratumoral bleeding followed by intraluminal rupture of a giant gastric GIST resulting in upper GI bleeding. The occurrence of intratumoral bleeding followed by rupture can be confirmed through a number of ways. First, by the macroscopic examination of the tumor, showing a large cystic cavity filled with blood that communicates to the stomach lumen. Secondly, by a reduction of the size of the tumor from 30 × 30 cm to 25 × 25 cm upon physical examination. Finally, by a significant decrease of hemoglobin levels less then 12 hours after the patient's admission. For these reasons, we believe that our patient presented intratumoral bleeding one day prior to admission, when the symptoms started, with subsequent rupture of a blood-filled tumoral cavity into the gastric lumen.

In conclusion, intratumoral bleeding is a very rare presentation of gastrointestinal stromal tumor; preoperative diagnosis is always difficult by the absence of pathognomonic signs or symptoms. The diagnosis should be suspect whenever there is a presentation of sudden abdominal pain in patients with an intraabdominal mass. Emergency local excision with negative margins associated with adjuvant therapy with KIT tyrosine kinase inhibitor remains the main modality of treatment for high risk gastrointestinal stromal tumors.

## Competing interests

The authors declare that they have no competing interests.

## Authors' contributions

RJCJ, RV, BMK and ALC participated in the admission and the care of this patient, the conception, manuscript preparation and literature search. LAC performed histopathological analyses and helped manuscript preparation. All authors read and approved the final manuscript.
